# Association of Standing Sacral–Abdominal Wall Distance and Android/Gynoid Fat Distribution with Locomotive Syndrome in Older Women

**DOI:** 10.3390/medicina62040674

**Published:** 2026-04-01

**Authors:** Takashi Nagai, Takako Tachikawa, Eriko Hoshi, Yu Nishida, Hanae Nomura, Mizuki Sugiyama, Fumihito Kasai, Yoshifumi Kudo, Nobuyuki Kawate

**Affiliations:** 1 Department of Rehabilitation Medicine, School of Medicine, Showa Medical University, Tokyo 142-8666, Japan; tachi.taka@med.showa-u.ac.jp (T.T.); eriko0215@med.showa-u.ac.jp (E.H.); yu9160n@med.showa-u.ac.jp (Y.N.); sugiyama-m@med.showa-u.ac.jp (M.S.); fumihito@med.showa-u.ac.jp (F.K.); kawate@med.showa-u.ac.jp (N.K.); 2 Department of Orthopaedic Surgery, School of Medicine, Showa Medical University, Tokyo 142-8666, Japan; kudo_4423@med.showa-u.ac.jp

**Keywords:** locomotive syndrome, sacral–abdominal wall distance, android/gynoid fat ratio, osteoporosis, older women

## Abstract

Background: Locomotive syndrome (LS) is a major cause of mobility impairment and loss of independence in older adults. Abdominal morphology and patterns of body fat distribution are thought to affect physical function; however, their relationships with the severity of LS in women with osteoporosis remain insufficiently characterized. Standing sacral–abdominal wall distance (SAD) is considered an indicator of compromised trunk support mechanisms, whereas the android/gynoid fat ratio (A/G ratio) primarily reflects body fat distribution patterns. Methods: This retrospective cross-sectional study included 221 women aged 65 years or older attending an osteoporosis outpatient clinic. LS was assessed using the stand-up test, the two-step test, and the Locomo 25 questionnaire according to official criteria. The most severe stage among the three tests was defined as the overall LS stage, and participants were classified into LS stage 0–1 and LS stage ≥ 2 groups. Associations of SAD and the A/G ratio with LS were examined using multivariable logistic regression analysis adjusted for age, skeletal muscle mass index (SMI), femoral neck T-score, and the Controlling Nutritional Status (CONUT) score. Results: Among the participants, 93 (42.1%) were classified as having LS stage ≥ 2. The LS stage ≥ 2 group had a significantly greater SAD than the LS stage 0–1 group (median, 167.3 mm vs. 154.6 mm; *p* < 0.001), whereas no significant difference was observed in the A/G ratio (*p* = 0.054). In multivariable analyses (n = 208), SAD was independently associated with LS stage ≥ 2 (odds ratio per 1 mm increase, 1.035; 95% confidence interval, 1.016–1.054; *p* < 0.001), corresponding to an odds ratio of 1.41 per 10 mm increase. The A/G ratio also showed an independent association, while no significant interaction between SAD and the A/G ratio was observed. Conclusions: In women with osteoporosis, SAD was independently associated with LS severity, reflecting mobility impairment from a perspective distinct from body fat distribution. SAD may serve as a simple and clinically useful indicator for assessing the risk of severe LS.

## 1. Introduction

Locomotive syndrome (LS) is defined as a state of declining mobility caused by dysfunction of the musculoskeletal system, which subsequently increases the likelihood of functional dependence and the need for long-term care [1]. In older women, LS progression is particularly affected by the combined presence of osteoporosis, reduced muscle strength, and age-related postural alterations [2,3,4]. In addition, sarcopenia has been increasingly recognized as a major contributor to mobility decline in older adults [5]. Aging-related loss of muscle mass and function affects motor unit integrity, trunk stability, and gait performance, thereby increasing vulnerability to mobility limitation [5]. Epidemiological studies have further demonstrated a substantial overlap between locomotive syndrome and sarcopenia, with many individuals exhibiting co-existence of these conditions [6]. Given the central role of trunk support in maintaining upright posture and efficient movement, the interaction between sarcopenia and abdominal morphological characteristics may further influence mobility capacity.

The development and progression of LS are thought to be associated not only with reduced muscle mass and decreased bone mineral density, but also with deterioration of trunk support mechanisms and changes in body morphology [4]. In recent years, sacral–abdominal wall distance (SAD) has gained attention as an index reflecting abdominal morphology [7]. Unlike conventional adiposity indices, SAD may capture decreased abdominal wall tension and impaired trunk support, rather than reflecting body fat accumulation alone [8]. Because trunk stability is essential for postural control and efficient movement, SAD may capture functional aspects of trunk support that are not adequately reflected by traditional indices of body composition.

In contrast, the android/gynoid fat ratio (A/G ratio) is a widely used indicator of body fat distribution and has been reported to be associated with metabolic abnormalities and cardiovascular risk [9,10]. However, its relationship with mobility impairment and the severity of LS has not been sufficiently investigated. Although both SAD and the A/G ratio are related to abdominal characteristics, they represent different physiological domains and may therefore show divergent associations with LS severity.

To date, few studies have simultaneously examined the relationships between SAD, the A/G ratio, and LS severity in women with osteoporosis. In particular, studies employing LS severity assessed according to official diagnostic criteria to clarify the clinical significance of these abdominal indices remain limited [11,12].

Therefore, the present study aimed to investigate the associations of SAD and the A/G ratio with the severity of locomotive syndrome in women aged 65 years or older attending an osteoporosis outpatient clinic, and to clarify the clinical relevance of abdominal morphological indices in the assessment of LS risk.

## 2. Materials and Methods

### 2.1. Study Design

**Figure 1:**
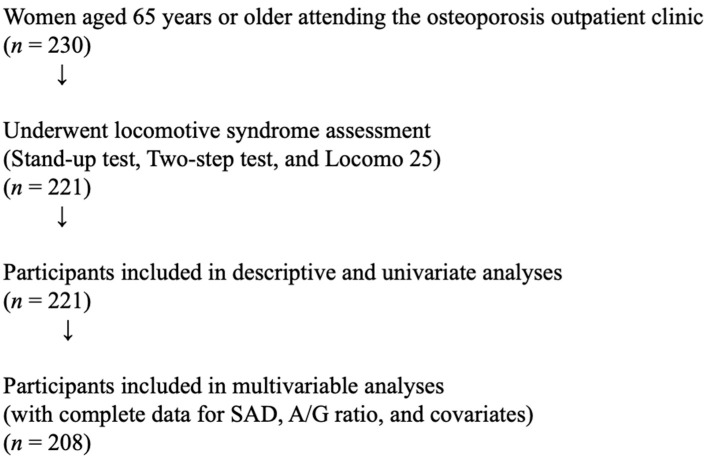
Flow chart of participant selection in the present study.

This study was conducted with approval from the Ethics Committee for Human Research at Showa Medical University (approval No. 2025-0459). Because of the retrospective study design, informed consent was obtained using an opt-out procedure. This was a single-center, retrospective, cross-sectional study. The study population comprised women aged 65 years or older who attended the osteoporosis outpatient clinic between April and December 2025. A total of 221 participants with complete data for locomotive syndrome assessment were included in the analyses. Among these, 208 participants with complete data for all covariates were included in the multivariable analyses. The participant selection process is shown in Figure 1.

### 2.2. Assessment of Locomotive Syndrome

Locomotive syndrome was assessed using three tests: the stand-up test, the two-step test, and the Locomo 25 questionnaire, in accordance with the official criteria proposed by the Japanese Orthopaedic Association [11]. Each test result was classified into four categories: non-applicable, locomotive syndrome stage 1, stage 2, and stage 3.

In this study, the most severe stage among the three tests was defined as the overall locomotive syndrome stage. For the primary analysis, overall locomotive syndrome stage ≥ 2 (locomotive syndrome stage ≥ 2) was used as the outcome, representing clinically meaningful mobility impairment, and comparisons were made with the stage 0–1 group. In analyses stratified by each locomotive syndrome test, the same threshold of locomotive syndrome stage ≥ 2 was applied.

### 2.3. Measurement of Sacral–Abdominal Wall Distance and Body Composition

Sacral–abdominal wall distance (SAD) was measured in the standing position using lateral whole-spine radiographs (Medical Systems USA, Inc., version 4.1.50107, New York, NY, USA) as the shortest distance between the anterior abdominal wall and the posterior margin of the superior endplate of the first sacral vertebra [13]. Measurements were performed twice, and the mean value was used for analysis. As an indicator of body fat distribution, the android/gynoid fat ratio (A/G ratio) was calculated using dual-energy X-ray absorptiometry (DXA) with a GE Healthcare Lunar Prodigy Advance system (enCORE software version 18; GE Healthcare, Chicago, IL, USA).

### 2.4. Covariates

Age, skeletal muscle mass index (SMI), and femoral neck bone mineral density T-score were included as clinical covariates. Nutritional status was evaluated using the Controlling Nutritional Status (CONUT) score [14]. Because the study population included users of both active vitamin D analogs and native vitamin D supplements, serum 25-hydroxyvitamin D [25(OH)D] levels were not used as a nutritional indicator in the analyses.

### 2.5. Statistical Analysis

Continuous variables are presented as medians and interquartile ranges, and categorical variables are presented as numbers and percentages. Statistical analyses were performed using Stat Flex software version 7.0.8 (Medical Watch Institute, Ube, Japan). Group comparisons were conducted using the Mann–Whitney U test. To identify factors associated with locomotive syndrome stage ≥ 2, multivariable logistic regression analysis was performed, and the results are presented as odds ratios with 95% confidence intervals. The interaction between SAD and the A/G ratio was also examined. Descriptive and univariate analyses were performed in all 221 participants, whereas multivariable analyses including SAD, the A/G ratio, and their interaction were conducted in 208 participants with complete data for these variables. A two-sided *p* value < 0.05 was considered statistically significant.

## 3. Results

### 3.1. Participant Characteristics and Distribution of Locomotive Syndrome Stages

When the most severe stage among the three locomotive syndrome tests was defined as the overall locomotive syndrome stage, 22 participants were classified as stage 0, 106 as stage 1, 41 as stage 2, and 52 as stage 3. Overall locomotive syndrome stage ≥ 2 was identified in 93 participants (42.1%), whereas 128 participants were classified as stage 0–1.

### 3.2. Comparison Between Locomotive Syndrome Stage 0–1 and Stage ≥ 2 Groups

A comparison of baseline characteristics between the locomotive syndrome stage 0–1 and stage ≥ 2 groups is shown in Table 1. Participants in the stage ≥ 2 group were significantly older than those in the stage 0–1 group (median age, 80 years [interquartile range, 76–85] vs. 75 years [70–80]; *p* < 0.001), and had significantly greater sacral–abdominal wall distance (SAD) (167.3 mm [151.2–187.2] vs. 154.6 mm [143.5–165.3]; *p* < 0.001).

Although the android/gynoid fat ratio (A/G ratio) was lower in the stage ≥ 2 group, the difference did not reach statistical significance (0.92 [0.72–1.06] vs. 1.00 [0.76–1.15]; *p* = 0.054). No significant differences were observed between the two groups in skeletal muscle mass index (SMI) or femoral neck T-score. The CONUT score tended to be higher in the stage ≥ 2 group; however, this difference was not statistically significant (*p* = 0.063) (Table 1).

### 3.3. Analyses According to Each Locomotive Syndrome Test

The results of comparisons between the locomotive syndrome stage 0–1 and stage ≥ 2 groups according to each locomotive syndrome test are shown in Table 2. In the two-step test, stand-up test, and Locomo 25, SAD was significantly greater in the stage ≥ 2 group in all tests (all *p* < 0.001). In contrast, the A/G ratio was not significantly associated with locomotive syndrome stage in any of the tests (Table 2).

### 3.4. Multivariable Analysis of Factors Associated with Locomotive Syndrome Stage ≥ 2

Multivariable logistic regression analysis was performed with overall locomotive syndrome stage ≥ 2 as the dependent variable (*n* = 208; Table 3). SAD was independently associated with locomotive syndrome stage ≥ 2 (odds ratio [OR] per 1 mm increase, 1.035; 95% confidence interval [CI], 1.016–1.054; *p* < 0.001). When SAD was scaled per 10 mm increase, the OR for locomotive syndrome stage ≥ 2 was 1.41 (95% CI, 1.17–1.70).

The A/G ratio also showed an independent association with locomotive syndrome stage ≥ 2 in the multivariable analysis (OR, 0.141; 95% CI, 0.034–0.583; *p* = 0.007). Age was significantly associated with locomotive syndrome stage ≥ 2, whereas SMI, femoral neck T-score, and CONUT score did not show independent associations.

### 3.5. Interaction Between SAD and A/G Ratio and Stratified Analysis

**Figure 2:**
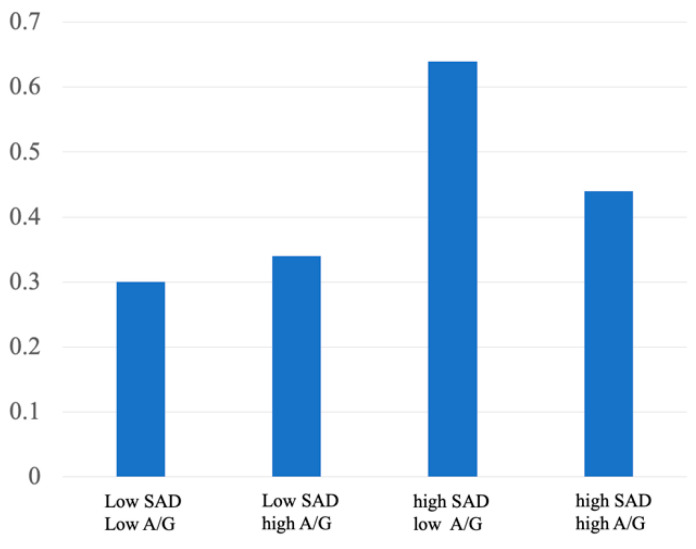
Proportion of participants with locomotive syndrome stage ≥ 2 according to sacral–abdominal wall distance (SAD) and android/gynoid fat ratio (A/G). Participants were classified using cut-off values of 160 mm for SAD and 1.0 for A/G ratio. A consistently higher proportion of locomotive syndrome stage ≥ 2 was observed in the high-SAD groups regardless of A/G ratio.

In analyses including the interaction term between SAD and the A/G ratio, no significant interaction was observed (*p* = 0.204). Furthermore, in stratified analyses in which participants were classified into four groups using cut-off values of 160 mm for SAD and 1.0 for the A/G ratio, the proportion of participants with locomotive syndrome stage ≥ 2 was consistently higher in the high-SAD groups, with minimal differences according to A/G ratio (Figure 2).

## 4. Discussion

**Figure 3:**
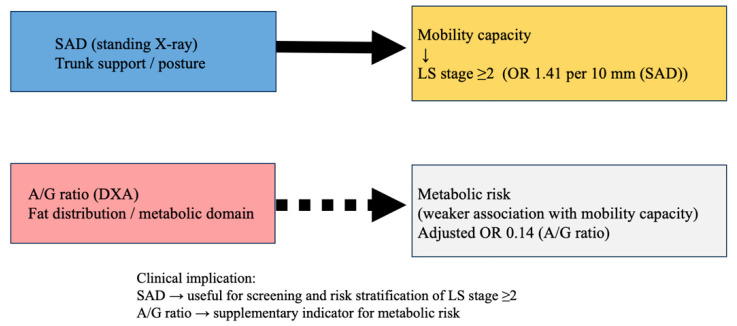
Conceptual framework illustrating the distinct physiological domains represented by sacral–abdominal wall distance (SAD) and the android/gynoid fat ratio (A/G ratio). SAD reflects trunk support and postural control, whereas the A/G ratio reflects fat distribution and the metabolic domain. In this study, locomotive syndrome (LS) severity was dichotomized into stage 0–1 and stage ≥ 2, the latter considered a clinically meaningful threshold associated with mobility impairment. SAD showed a stronger association with LS stage ≥ 2, suggesting that trunk support may be more directly linked to mobility than fat distribution itself.

In this study, we investigated the associations of abdominal morphological indices—namely sacral–abdominal wall distance (SAD) and the android/gynoid fat ratio (A/G ratio)—with the severity of locomotive syndrome (LS) in women aged 65 years or older attending an osteoporosis outpatient clinic. The main finding of this study was that SAD showed an independent association with LS stage ≥ 2 after adjustment for age, skeletal muscle mass index, femoral neck bone mineral density, and nutritional status. This association was consistently observed across all three LS assessment tools, supporting the robustness of the relationship between SAD and LS severity (Figure 3).

SAD, when measured in the standing position, reflects the anteroposterior dimension of the abdomen and has been proposed to represent not only abdominal fat accumulation but also reduced abdominal wall tension and impairment of trunk support mechanisms [8]. Unlike conventional anthropometric or body composition indices, SAD may capture postural and morphological characteristics related to trunk stability. Given the essential role of trunk support in balance regulation, transitional movements, and gait performance, impairment of this function may substantially contribute to mobility limitations [4,13]. The present findings support the clinical relevance of SAD as a marker associated with LS severity from a perspective distinct from muscle mass or bone density alone. Although waist circumference (WC) has been shown to correlate with SAD and is easier to obtain in clinical practice, it primarily reflects abdominal girth and fat accumulation. In contrast, standing SAD measured under weight-bearing conditions captures the anteroposterior abdominal dimension in relation to sagittal spinal alignment and trunk mechanics. Thus, SAD may provide complementary information regarding postural support and weight-bearing balance that is not fully reflected by conventional anthropometric measures alone.

This interpretation is further supported by the stratified analysis shown in Figure 2, in which a consistently higher prevalence of LS stage ≥ 2 was observed in the high-SAD groups regardless of the A/G ratio. In contrast, the A/G ratio, a widely used indicator of body fat distribution and metabolic risk [15,16], showed a weaker association with LS severity in univariate analyses. Although the A/G ratio demonstrated an independent association with LS stage ≥ 2 in the multivariable model, its contribution appeared secondary to that of SAD. Furthermore, no significant interaction between SAD and the A/G ratio was observed, suggesting that the association between SAD and LS severity was not modified by body fat distribution. These findings suggest that trunk morphology and support-related characteristics may have a stronger association with mobility impairment than fat distribution per se. In the present study, the inverse association observed between the A/G ratio and LS stage ≥ 2 in the fully adjusted multivariable model warrants careful interpretation. Because the study population consisted of older women attending an osteoporosis outpatient clinic, with BMI values largely within the normal range, a relatively higher A/G ratio may not necessarily indicate excess adiposity but rather comparatively preserved body mass within a generally lean cohort. In this context, lower A/G values may reflect reduced physiological reserve or frailty-related characteristics rather than favorable body composition. Moreover, LS primarily reflects impairment of trunk support and mobility capacity rather than metabolic risk per se. Therefore, the observed inverse association should not be interpreted as evidence of a protective causal effect of a higher A/G ratio, but rather as reflecting population-specific characteristics and potential statistical adjustment effects.

In this study, LS severity was dichotomized into stage 0–1 and stage ≥ 2. Although LS is inherently an ordinal construct ranging from stage 0 to stage 3, LS stage ≥ 2 is generally regarded as a clinically meaningful threshold indicating substantial decline in mobility and increased risk of functional limitation [17,18]. The dichotomization was therefore adopted to enhance clinical interpretability and applicability. Nevertheless, this approach inevitably involves loss of ordinal information and may obscure differences between adjacent stages, such as between stage 1 and stage 2 or between stage 2 and stage 3. Accordingly, the results should be interpreted with consideration of potential cutoff dependency.

Overall LS stage was defined as the most severe stage among the stand-up test, two-step test, and Locomo 25 questionnaire. This approach increases sensitivity for detecting mobility impairment but may overestimate functional decline, particularly because the Locomo 25 includes subjective components such as pain, anxiety, and perceived difficulty. These subjective elements may be influenced by postural abnormalities or trunk discomfort and could potentially strengthen the observed association with SAD. However, the consistent association between SAD and LS severity across all three individual tests suggests that the findings are not solely driven by a single assessment method.

It is also important to distinguish between mobility capacity and actual daily physical activity. The LS assessments used in this study primarily evaluate mobility capacity and perceived difficulty rather than real-world activity performance, such as step counts or objectively measured physical activity levels. Therefore, the present results should be interpreted as indicating an association between SAD and a state of increased risk for activity limitation, rather than a direct reduction in actual daily activity. From a rehabilitation perspective, this distinction is essential to avoid overinterpretation of the findings.

In this study, the CONUT score was used as an indicator of nutritional status. Because the study population included users of both active vitamin D analogs and native vitamin D supplements, serum 25-hydroxyvitamin D levels were considered susceptible to the effects of supplementation and were therefore not included in the analyses [19,20]. The use of the CONUT score allowed a more comprehensive adjustment for nutritional status, which represents a strength of the present study.

Several limitations should be acknowledged. First, the retrospective cross-sectional design precludes causal inference, and SAD should be regarded as a risk stratification marker rather than a predictive or causal factor. Second, SAD reflects abdominal and trunk morphology but does not directly assess trunk muscle strength or motor control. In addition, sagittal spinal alignment parameters were not included as covariates, and thus SAD may partly act as a surrogate marker for spinal deformity or postural changes. Third, important potential confounders such as physical activity level, lifestyle factors, and exercise habits were not evaluated, and residual confounding cannot be excluded. Additionally, BMI values in the present cohort were generally within the normal range, reflecting the characteristics of patients attending an osteoporosis outpatient clinic. In populations with a higher prevalence of overweight or obesity, absolute SAD values and optimal cutoff thresholds for LS risk stratification may differ. Therefore, the generalizability of specific SAD thresholds requires validation in cohorts with diverse body composition profiles. Longitudinal studies incorporating objective activity measures and detailed postural assessments are warranted to clarify the temporal relationship between SAD and LS progression, as well as the potential modifiability of SAD through targeted interventions.

Despite these limitations, the present study has several strengths, including the use of official LS diagnostic criteria, consistent findings across multiple LS assessment tools, and adjustment for key clinical covariates in a relatively large cohort of older women with osteoporosis. These features enhance the clinical relevance and interpretability of the results.

In summary, SAD was independently associated with LS severity in older women with osteoporosis, reflecting mobility impairment from a perspective distinct from conventional body fat distribution indices. SAD may serve as a simple and clinically useful marker for identifying individuals at higher risk of significant mobility limitation, thereby contributing to early risk stratification and the development of targeted preventive strategies.

## 5. Conclusions

Consistent with the results of the multivariable analyses, in this study of women aged 65 years or older attending an osteoporosis outpatient clinic, sacral–abdominal wall distance (SAD) was independently associated with the severity of locomotive syndrome, defined as LS stage ≥ 2 according to official diagnostic criteria. This association was observed after adjustment for age, skeletal muscle mass index, femoral neck bone mineral density, and nutritional status, and was consistently identified across multiple locomotive syndrome assessment tools.

Importantly, SAD should be interpreted as a marker associated with an increased risk of clinically meaningful mobility limitation rather than as a causal or predictive factor. The present findings reflect mobility capacity and perceived functional difficulty rather than actual daily physical activity performance. In this context, SAD captures aspects of trunk morphology and support-related characteristics that are not fully explained by conventional indices of body fat distribution, such as the android/gynoid fat ratio.

Taken together, these results suggest that SAD may function as a simple and clinically applicable marker for identifying individuals at increased risk of clinically meaningful mobility limitation. Further longitudinal and interventional studies incorporating objective measures of physical activity and postural alignment are required to clarify the temporal relationship and clinical utility of SAD in the prevention and management of locomotive syndrome.

## Figures and Tables

**Table 1:** Baseline characteristics of participants according to locomotive syndrome severity.

Variable	Overall (*n* = 221)	Locomotive Syndrome Stage 0–1 (*n* = 128)	Locomotive Syndrome Stage ≥ 2 (*n* = 93)	*p*-Value *
Age (years)	77 [72–83]	75 [70–80]	80 [75–84]	<0.001
BMI (kg/m^2^)	21.0 [19.1–23.2]	21.0 [18.9–22.8]	21.5 [19.4–23.8]	0.142
Sacral–abdominal wall distance (SAD, mm)	158.4 [146.4–172.7]	154.6 [143.5–165.3]	167.3 [151.2–187.2]	<0.001
Android/gynoid fat ratio (A/G ratio)	0.98 [0.75–1.13]	1.00 [0.76–1.15]	0.92 [0.72–1.06]	0.054
Skeletal muscle mass index (SMI, kg/m^2^)	5.61 [5.10–6.07]	5.56 [5.10–5.96]	5.65 [5.11–6.25]	0.210
Femoral neck T-score	−2.40 [−2.80–1.85]	−2.40 [−2.75–1.90]	−2.40 [−2.80–1.80]	0.858
CONUT score	1 [0–2]	0.5 [0–2]	1 [0–2]	0.063
Charlson Comorbidity Index (CCI)	0 [0–1.5]	0 [0–1]	0 [0–2]	0.184
Frailty score (0–3)	1 [0–2]	1 [0–1]	1 [1–2]	<0.001

Values are presented as median [interquartile range]. * *p*-values were calculated using the Mann–Whitney U test for comparisons between locomotive syndrome stage 0–1 and stage ≥ 2.

**Table 2:** Associations of sacral–abdominal wall distance and android/gynoid fat ratio with locomotive syndrome severity according to each locomotive syndrome test.

(a) Two-Step Test			
**Variable**	**Locomotive Syndrome Stage 0–1 (** ***n*** ** = 153)**	**Locomotive Syndrome Stage ≥ 2 (** ***n*** ** = 68)**	***p*** **-Value**
Sacral–abdominal wall distance (SAD, mm)	156.1 [145.0–166.8]	168.3 [151.2–191.7]	<0.001
Android/gynoid fat ratio (A/G ratio)	1.00 [0.75–1.15]	0.92 [0.76–1.05]	0.080
(b) Stand-Up Test			
**Variable**	**Locomotive Syndrome Stage 0–1 (** ***n*** ** = 166)**	**Locomotive Syndrome Stage ≥ 2 (** ***n*** ** = 53)**	***p*** **-Value**
Sacral–abdominal wall distance (SAD, mm)	156.1 [144.6–166.8]	170.4 [153.4–192.2]	<0.001
Android/gynoid fat ratio (A/G ratio)	0.98 [0.75–1.13]	0.98 [0.82–1.13]	0.902
(c) Locomo 25			
**Variable**	**Locomotive Syndrome Stage 0–1 (** ***n*** ** = 167)**	**Locomotive Syndrome Stage ≥ 2 (** ***n*** ** = 53)**	***p*** **-Value**
Sacral–abdominal wall distance (SAD, mm)	156.1 [144.8–167.0]	169.9 [156.9–192.7]	<0.001
Android/gynoid fat ratio (A/G ratio)	1.00 [0.76–1.14]	0.90 [0.71–1.04]	0.056

Values are presented as median [interquartile range]. *p*-values were calculated using the Mann–Whitney U test for comparisons between locomotive syndrome stage 0–1 and stage ≥ 2.

**Table 3:** Multivariable logistic regression analysis for factors associated with locomotive syndrome stage ≥ 2.

Variable	Odds Ratio (OR)	95% Confidence Interval	*p*-Value
Sacral–abdominal wall distance (SAD, per 1 mm increase)	1.035	1.016–1.054	<0.001
Android/gynoid fat ratio (A/G ratio)	0.141	0.034–0.583	0.007
Age (per 1-year increase)	1.132	1.073–1.194	<0.001
Skeletal muscle mass index (SMI, kg/m^2^)	0.730	0.432–1.234	0.24
Femoral neck T-score	1.222	0.764–1.954	0.40
CONUT score	1.170	0.863–1.586	0.31

Akaike Information Criterion (AIC): 240.18, Area Under the Curve (AUC): 0.778. Odds ratios were estimated using a multivariable logistic regression model.

## Data Availability

The datasets generated and/or analyzed during the current study are available from the corresponding author on reasonable request.

## Abbreviations

The following abbreviations are used in this manuscript:

A/G	Android adiposity/Gynoid adiposity
CCI	Charlson Comorbidity Index
CONUT	Controlling Nutritional Status
DXA	Dual-energy X-ray Absorptiometry
LS	Locomotive syndrome
SAD	Sacral-abdominal wall distance
SMI	Skeletal Muscle Index
25(OH)D	25-hydroxyvitamin D
